# Defining adequate contact for transmission of *Mycobacterium tuberculosis* in an African urban environment

**DOI:** 10.1186/s12889-020-08998-7

**Published:** 2020-06-09

**Authors:** María Eugenia Castellanos, Sarah Zalwango, Robert Kakaire, Mark H. Ebell, Kevin K. Dobbin, Juliet Sekandi, Noah Kiwanuka, Christopher C. Whalen

**Affiliations:** 1 Global Health Institute, College of Public Health, University of Georgia, Athens, Georgia; 2 Department of Epidemiology and Biostatistics, College of Public Health, University of Georgia, Athens, Georgia; 3 College of Health Sciences, School of Public Health, Makerere University, Kampala, Uganda

**Keywords:** Tuberculosis, Contact, Transmission, Social network, Contact network, Social mixing

## Abstract

**Background:**

The risk of infection from respiratory pathogens increases according to the contact rate between the infectious case and susceptible contact, but the definition of adequate contact for transmission is not standard. In this study we aimed to identify factors that can explain the level of contact between tuberculosis cases and their social networks in an African urban environment.

**Methods:**

This was a cross-sectional study conducted in Kampala, Uganda from 2013 to 2017. We carried out an exploratory factor analysis (EFA) in social network data from tuberculosis cases and their contacts. We evaluated the factorability of the data to EFA using the Kaiser-Meyer-Olkin Measure of Sampling Adequacy (KMO). We used principal axis factoring with oblique rotation to extract and rotate the factors, then we calculated factor scores for each using the weighted sum scores method. We assessed construct validity of the factors by associating the factors with other variables related to social mixing.

**Results:**

Tuberculosis cases (N = 120) listed their encounters with 1154 members of their social networks. Two factors were identified, the first named “Setting” captured 61% of the variance whereas the second, named ‘Relationship’ captured 21%. Median scores for the setting and relationship factors were 10.2 (IQR 7.0, 13.6) and 7.7 (IQR 6.4, 10.1) respectively. Setting and Relationship scores varied according to the age, gender, and nature of the relationship among tuberculosis cases and their contacts. Family members had a higher median setting score (13.8, IQR 11.6, 15.7) than non-family members (7.2, IQR 6.2, 9.4). The median relationship score in family members (9.9, IQR 7.6, 11.5) was also higher than in non-family members (6.9, IQR 5.6, 8.1). For both factors, household contacts had higher scores than extra-household contacts (*p* < .0001). Contacts of male cases had a lower setting score as opposed to contacts of female cases. In contrast, contacts of male and female cases had similar relationship scores.

**Conclusions:**

In this large cross-sectional study from an urban African setting, we identified two factors that can assess adequate contact between tuberculosis cases and their social network members. These findings also confirm the complexity and heterogeneity of social mixing.

## Background

For any infectious disease, the transmission of the microorganism is the result of exposure, that is adequate contact between an infectious case and a susceptible host. The nature of adequate contact varies from one infectious disease to another, but regardless of the disease, the definition of adequate contact is not standardized. This lack of definition is especially true for airborne infectious diseases [1–3] for which the sequence, frequency, and duration of contact is challenging to ascertain [4]. Nevertheless, the rate of adequate contact is an important parameter to understand because it is a key determinant of epidemic behavior [5]. Without a clear understanding of the factors that affect the contact rate, it may not be possible to design effective community interventions to minimize transmission.

Tuberculosis is a respiratory disease that is transmitted through the airborne route. Although the global incidence of tuberculosis has declined in the last decade, the disease persists in many low- and middle-income countries around the world [6]. These countries rely on passive case finding followed by directly-observed therapy as the mainstay of tuberculosis control, yet the disease persists. New approaches are needed. We propose that community-based interventions that reduce the frequency and duration of adequate contact between tuberculosis cases and their contacts would be an effective way to control tuberculosis, but it is challenging to design these interventions without a better understanding of the factors that affect the dynamics of social mixing in a population.

From previous studies of tuberculosis and other respiratory pathogens, we know that demographics and the settings of interaction may modify the nature of contact between cases and their contacts [1, 3, 7–10]. Age assortment is observed in the community, whereas intergenerational mixing is found within households. In general, individuals may have contact with members of their social networks in a limited number of settings, such as home, school, and workplace [11–14]. At best, this understanding of social network mixing and movement in the community give an incomplete description of adequate contact for transmission of *M. tuberculosis*. To develop a more complete understanding of adequate contact, we performed an exploratory factor analysis (EFA) using social network data collected from index tuberculosis cases and their contacts as identified in their ego-centric social networks, that is the list of personal networks defined by the index case [15]. We chose to use EFA because it is a method that can synthesize correlated data [16] into underlying constructs called factors [17], thereby reducing the data into meaningful concepts that provide insight into the nature of adequate contact. We also evaluated the construct validity of the factors we identified by evaluating their association with other variables related to social mixing.

## Methods

We conducted a cross-sectional study of patients with active tuberculosis in Kampala, Uganda, from July 2013 to February 2017. The burden of tuberculosis is very high in Uganda (200 new cases per 100,000 population) and is especially high in urban settings [6]. In Kampala, the prevalence of disease has been estimated between 440 and 800 cases per 100,000 [18, 19]. Index cases for this study were recruited from the Ugandan National Tuberculosis and Leprosy Programme through its referral centers at Mulago Hospital and Lubaga Hospital and through a network of community clinics operated by the Kampala Capital City Authority. Eligible index cases were those with microbiologically confirmed, pulmonary tuberculosis, 15 years or older, residents of Lubaga Division of Kampala, who gave written informed consent to participate in the study. We excluded tuberculosis patients treated for more than a week with antituberculosis medication. The sample size was determined based on the number of tuberculosis cases and paired controls needed to compare the prevalence of tuberculous infection among their household and extra-household contacts (power = 80%, error = 5%, estimated difference in tuberculous infection = 6%).

In a standard interview conducted by trained field workers, index cases provided a list of their household and community members (defined as ‘extra-household’) of their ego-centric networks. To reduce recall bias, the interviewers used a combination of name and location generators, or standard prompts, and recent timeframes to help participants remember their household and extra-household members. For our study, a contact was defined as a person with whom the index case had a personal relationship, such as family, relative, friends, and work partners [2, 20]. Household contacts were those that resided in the household of an index case for the previous 3 months and had eaten meals in the household at least weekly [21, 22]; otherwise, contacts were classified as extra-household. Once enrolled, the social network members were interviewed using a standard questionnaire used in previous studies in Uganda to collect epidemiologic information relevant to the nature and duration of contact in their social networks [23, 24] (Supplementary Material, Table S1).

An item analysis was conducted in the original data set to determine baseline characteristics of the tuberculosis cases, explore the distribution of each variable, and summarized proportions and measures of central tendency. After assessing the distributions, we combined and redefined variables as dichotomous or ordinal to ensure a similar scale across variables and to assign values in which the lowest value represented less contact and the highest value represented the more contact.

For the EFA, we selected 15 variables. These variables were length of relationship, change in the frequency of contact since onset of cough, how well case knows contact, trust between case and contact, case confided with contact about tuberculosis diagnosis, frequency and duration of contact in last month, shared meals, sleeping together, care provided to case by contact, place of usual meeting with contact, meeting venue as indoors or outdoors, ventilation of usual meeting, number of people at usual location, means of transportation used most often with contact, and knowledge of contact having cough (Supplementary Material, Table S2**)**. We did not consider for EFA variables that were nominal, had limited distribution, or did not provide additional content (Supplementary Material, Table S3 & Fig. S1**)**.

**Fig. 1:**
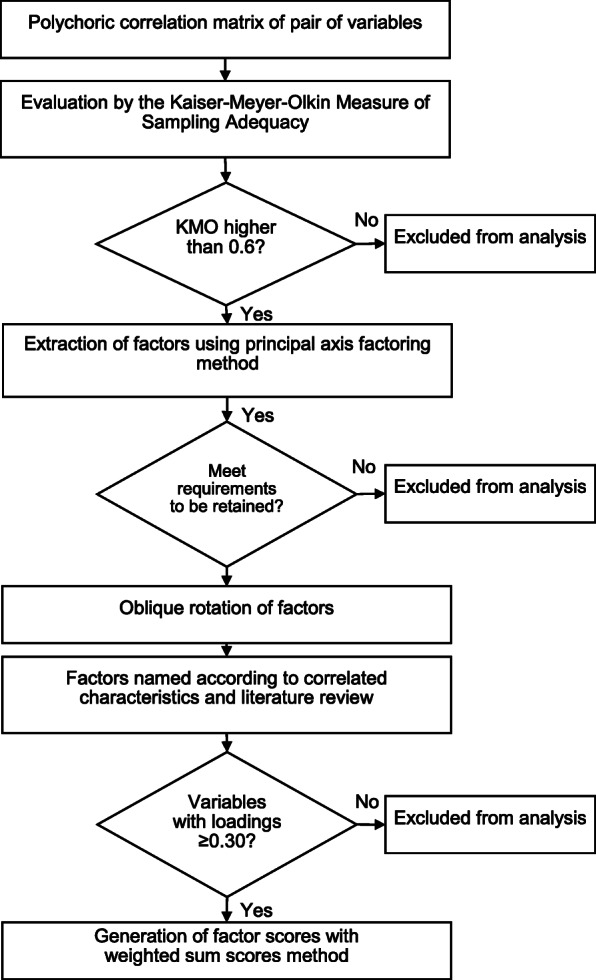
Flow chart of the process for exploratory factor analysis in the study

We then performed an EFA to determine the underlying factors (Fig. 1). By accounting for correlations among variables, this method allowed us to reduce the number of variables needed to define adequate contact. We chose EFA over principal component analysis since we hypothesized that the observed variables could be grouped in underlying constructs, or factors [25], that would provide meaningful insights into the nature of adequate contact. To begin, we estimated the polychoric correlation between all pairs of the 15 variables to assess the relations among them [26]. We next evaluated the factorability of these variables using the Kaiser-Meyer-Olkin Measure of Sampling Adequacy (KMO) [27]. Of the 15 variables, 13 had a KMO measure of 0.6 or greater and were included in the next step [28]. Once the correlation matrix was estimated, we used the principal axis factoring method to extract the factors [17] and retained factors based on proposed criteria [29]: 1) factors with eigenvalue greater than 1, 2) factors with eigenvalues that appear in the sharp slope of a Scree plot, and 3) factors that account for 80–90% of the variation. Moreover, only factors with at least three variables were retained [30]. Since the factors were likely correlated, we performed an oblique rotation of the factors [31] to arrive at the final set of factors. We then assigned a name to each factor based on their correlated characteristics and literature review [32].

To generate the factor scores for an individual, we used the weighted sum scores method, as it allows that the variables with the highest loadings to have the highest impact in our factor score [33]. To compute factor scores for each study participant, we first multiplied the recorded value for each item by the corresponding estimated factor loading then summed these products across all variables to generate the factor score. We excluded variables with loadings below 0.30 [34]. Factor scores were investigated to determine whether they met the normality assumption and had a unimodal distribution [35]. Based on these analyses, the results of the factor scores are presented as median with interquartile ranges.

To establish the construct validity of the factor scores, we examined the relationship of each factor with relevant variables in a sub-set of the study participants. We stratified these participants according to type of contact (household and extra-household contact) and nature of the relationship between case and control (spouse, child, sibling, friend, co-worker, other relatives, neighbor, other). The median and interquartile ranges of the factor scores were estimated for each stratum. In the process, we examined the variability of the factor scores by gender of contact, age of contact, age of index and sex of index case. To compare the difference in medians among stratified groups we estimated the 95% confidence intervals by bootstrapping, using the package ‘boot’ for R software [36]. We set the number of bootstraps replicates to 10,000 and calculated the intervals with the adjusted bootstrap percentile (BCa) method [37]. We also examined the relationship of the factor scores with demographic characteristics and household exposure using a multivariate linear regression model.

Written informed consent was obtained from all participants prior to study inclusion. Institutional review board clearance was obtained from the Higher Degrees and Ethics Committee at Makerere University School of Public Health, the Uganda Council of Science and Technology, and the University of Georgia.

## Results

The study enrolled 120 index cases with tuberculosis disease and 1179 household and community contacts to generate social networks. Complete social network information was obtained for 1154 of 1179 contacts (98%) and 940 of them had full demographic information. The majority of index cases was men (83%), young adults (57%) and had a microbiologically confirmed diagnosis (Table 1). Each case had a median of nine contacts (IQR 8, 12). For male cases, the median number of household contacts was 4 (IQR 2,6) and extra-household contacts was 7 (IQR 4,9), whereas for women, the median number of household contacts was also 4 (IQR 2,6), and extra-household contacts was 5.5 (IQR 4,8). The median age of the contacts was 23 years (IQR 13–32), they had a similar distribution in terms of sex and 62% of them were community contacts (Table 2).

**Table 1:** Baseline characteristics of index cases who provided social network information

Category	No.	(%)
**Total number of index cases**	**120**	
Male gender	83	69
Age, years, median [IQR^1^]	28 [23–36]	
**Age (category)**		
15–24	38	32
25–44	68	57
45 or more	14	12
**Tribe**		
Ganda	86	72
Nyakitara	4	3
Lunyankole	12	10
Lusoga	2	2
Other	14	12
Missing	2	2
**TB diagnosis**		
Microscopy and culture	109	91
Only microscopy	5	4
Only culture	4	3
Only X-ray/clinical diagnosis	2	2

^**1**^ Interquartile range

**Table 2:** Characteristics of the 940 contacts with a full complement of demographic information from Kampala, Uganda that answered the social network survey

Characteristic	N	%
**Gender**		
Male	462	49
Female	478	51
**Type of contact**		
Household	350	37
Extra-household	590	66
**Age, years, median [IQR]**	23 [13–32]	
**Age (category)**		
0–14	245	26
15-greater	695	74
**Residence**		
Lives in Rugaba	905	96
Do not live in Rugaba	34	4
No information available	01	0
**HIV Result**		
Positive	71	8
Negative	851	91
No information available	18	2
**BCG vaccine**		
Yes (verbal report/immunization card)	803	85
No prior vaccination	80	8
Don’t’ know/Missing	57	6

The overall KMO of the thirteen variables considered for EFA was 0.72, with individual KMO measurements of > 0.60 (Table 3). Two factors explained 82% of the variance (61 and 21% respectively) and met our selection criteria. The factor loading of the first factor was 5.7 and for the second factor 1.9 (Supplementary material, Fig. S2).

**Table 3:** Individual and Overall Kaiser’s Measure of Sampling Adequacy. Initial results with 15 variables and final selection with 13 variables included in the exploratory factor analysis

Variable	Kaiser’s Measure of Sampling Adequacy 15 items	Kaiser’s Measure of Sampling Adequacy 13 items
**Overall**	**0.55**	**0.72**
Contact happen indoors or outdoors	0.50	0.61
Nature of ventilation at usual place of meeting	0.47	0.63
Case shared TB diagnosis with contact	0.50	0.69
Contact have cough	0.55	0.69
Frequency of shared meals since onset cough	0.56	0.72
Frequency and duration of contact over the past month	0.82	0.73
Care was provided by the contact in the past 3 months	0.54	0.73
Place of usual meeting. Home TB case versus other location.	0.80	0.75
Case trusts contact	0.66	0.75
Length of knowing contact	0.71	0.77
Frequency of sleeping in same room and bed since onset cough	0.55	0.79
How well does the case knows contact	0.55	0.80
Means of transportation used most often with contact. None (walking) versus a type of transportation.	0.80	0.81
Frequency of meeting since onset cough	0.37	NE^1^
Number of other people met in addition to contact	0.12	NE

^1^Not estimated as it was not included in the exploratory factor analysis

The first factor grouped together variables related to the setting and environment of the contact between the index case and his/her contact; we named this factor the “setting” factor. The six variables in this category had factor loadings of 0.60 or more (Table 4). The second factor grouped together variables that corresponded to the intimacy and social relationship of the index case and contact; we named the factor the “relationship” factor. The six variables in this category had factor loadings of 0.50 or more (Table 4). Variable “Contact has cough” produced low factor loadings in both factors, implying that this variable might not contribute particularly to either of them.

**Table 4:** Factor loadings matrix identified by exploratory factor analysis when two factors were retained. Bold font indicates variables that are grouped in each factor

Variable	Factor1 (Setting)	Factor2 (Relationship)
Nature of ventilation at usual place of meeting	**0.8247**	−0.10068
Frequency of sleeping in same room and bed since onset cough	**0.81413**	0.07244
Contact happen indoors or outdoors	**0.8065**	−0.05468
Frequency of shared meals since onset cough	**0.76773**	0.19686
Place of usual meeting. Home TB case versus Other location.	**0.71749**	−0.04231
Frequency and duration of contact over the past month	**0.62832**	0.10417
Case trusts contact	−0.20248	**0.94816**
Case shared TB diagnosis with contact	−0.13705	**0.92354**
Care was provided by the contact in the past 3 months	0.24285	**0.72162**
Length of knowing contact	0.21973	**0.55998**
How well does the case knows contact	0.38398	**0.52836**
Means of transportation used most often with contact. None (walking) versus a type of transportation.	0.08259	**0.50289**
Contact have cough	0.03227	0.15773

**Fig. 2:**
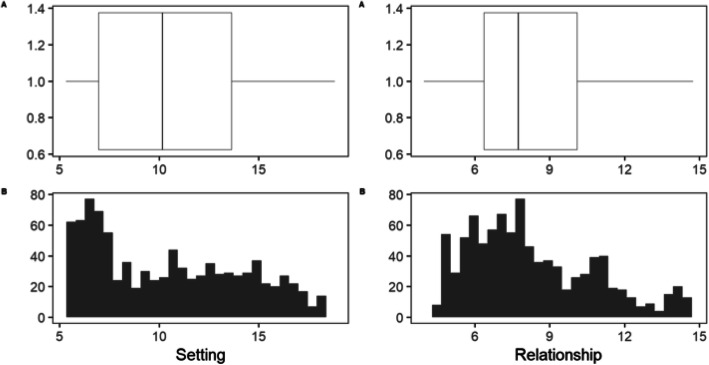
Distribution of closeness factors among the study population (n = 940). A histogram and a boxplot are shown to study the distribution of the Setting and Relationship factor. Left Panel: Setting Factor. Right Panel: Relationship Factor

**Fig. 3:**
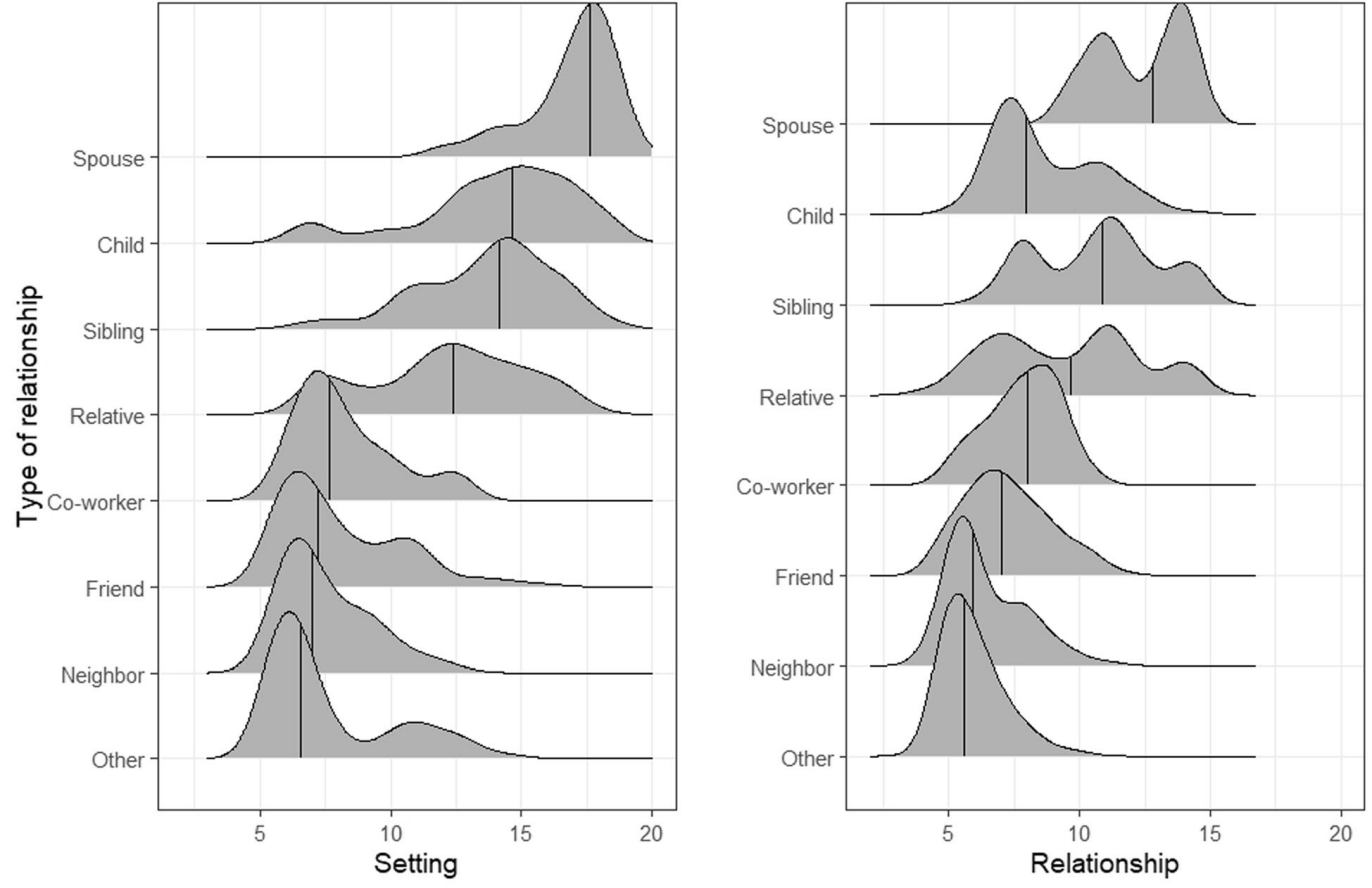
Distribution of the Setting and Relationship factors according to the nature of the relationship between a tuberculosis case and their contacts (n = 940). A ridgeline plot is shown to study the distribution of the Setting and Relationship factor scores, according to the nature of relationship between tuberculosis case and contact. Vertical lines indicate the median value for each group. Left Panel: Setting Factor. Right Panel: Relationship Factor

Among the 940 contacts with a full complement of information, we generated setting and relationship scores. In this subset of contacts, the social network findings corresponded well to the findings obtained from the full set of 1154 contacts (Table S4), thereby reducing the likelihood of selection bias. Both the setting factor and relationship factor followed a multimodal distribution (*p* = 0.02 and *p* < 0.0001, respectively; unimodality test) (Fig. 2). Scores for the setting factor had a median of 10.2 (IQR 7.0, 13.6) with a range of 5.3–18.8; the relationship scores had a median of 7.7 (IQR 6.4, 10.1) with a range of 4.0 to 14.8. Setting and relationship scores varied according to the nature of the relationship among a tuberculosis case and their contact (Fig. 3). Spouses had the highest setting score, followed by children and siblings. Altogether, family members had a higher median setting score (13.8, IQR 11.6, 15.7) than non-family members (7.2, IQR 6.2, 9.4). In the case of the relationship factor, spouses, siblings, and other relatives had the highest score. The median relationship score in family members (9.9, IQR 7.6, 11.5) was also higher than in non-family members (6.9, IQR 5.6, 8.1).

**Fig. 4:**
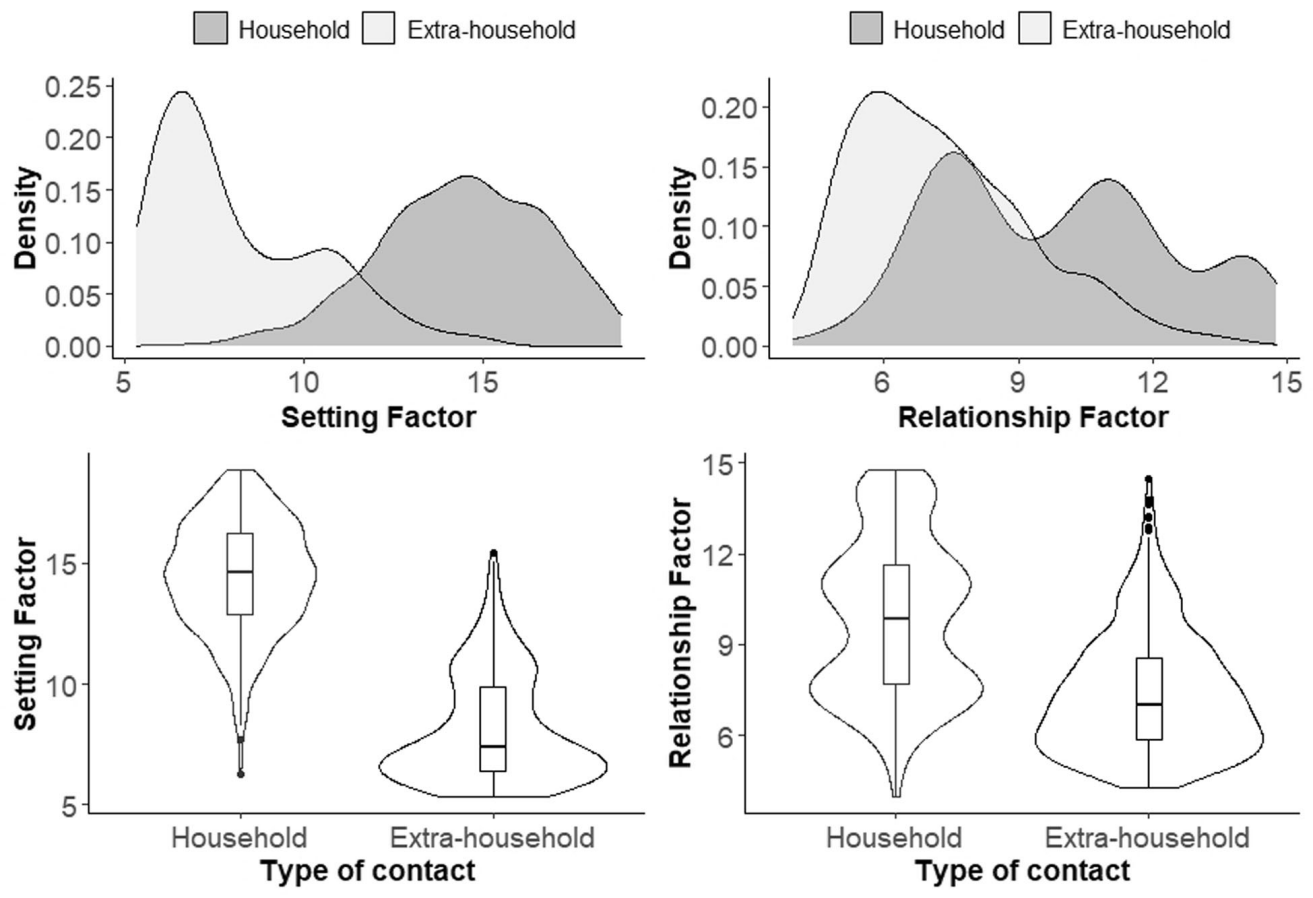
Distribution of the Setting and Relationship factors among household and extra-household contacts (n = 940). A histogram and a violin plot are presented to describe the distribution of the Setting and Relationship Factor Scores among household and extra-household contacts of the tuberculosis case. The values on the X-axis of the histogram indicates the score of each factor. The Y-axis indicates the relative frequency (density) at each Factor score. Inside each violin plot a box plot is presented. Left panel: Histogram (top) and violin plot (bottom) for the Setting Factor. Right panel: Histogram (top) and violin plot (bottom) for the Relationship Factor

For both the setting and relationship scores, household contacts (N = 350) had higher scores than extra-household contacts (*N* = 590, Fig. 4, *p* < .0001). For the setting factor, household contacts had a greater median score (14.6, IQR 12.8,16.2) as compared to extra-household contacts (7.4, IQR 6.4, 9.8); the difference in medians was 7.2 (95% CI: 6.9, 7.6). For the relationship factor, household contacts again had a greater median score (9.8, IQR 7.7, 11.6) as compared to extra-household contacts (7.0, IQR 5.8, 8.6). The difference in medians was 2.8 (95% CI 2.2–3.5). Extra-household family members had a higher median setting score (9.9, IQR 7.3, 11.8) than extra-household non-family members (7.1, IQR 6.2, 9.1). Similarly, extra-household family members had a higher median relationship score (9.4, IQR 7.0,11.2) than extra-household non-family members (6.8, IQR 5.6, 8.1).

When stratifying the analysis by the sex or age of the index case, the median values of the factors scores among contacts revealed differences according to age and sex of the participants (Table 5). Contacts of male cases had a lower setting score as compared to contacts of female cases (difference in medians = 2.2 (95% CI 1.4–3.3)). In contrast, contacts of male and female cases had similar relationship scores. These observations were confirmed in a multivariable regression analysis (Supplementary Material, Table S5 & Table S6). Contacts ≤4 years old had the highest setting score, followed by contacts aged 5–14 years and contacts ≥15 years old. The difference in medians among contacts ≤15 years of age and contacts ≥15 years of age was 4.6 (95% CI 3.7, 5.8). Interactions of contacts ≥15 years old with cases aged 25–44 years were the ones with the lowest setting score. In contrast, interactions of contacts ≤15 years with cases 15–24 years proved to be the highest**.** For the relationship score, an inverse association with the age of contact was found.

**Table 5:** Median Setting and Relationship scores (with interquartile range-IQR) from the sub-set of 940 enrolled contacts with demographic variables collected. Overall and stratified by gender and age of cases and contacts

Variable	N (%)	Setting Score Median (IQR)	Relationship Score Median (IQR)
**Overall**	940 (100)	10.2 (7.0,13.6)	7.7 (6.4,10.1)
**Stratified by Gender Index**			
**Contact with Male Index**	661	9.3 (6.8,12.9)	7.8 (6.2,10.1)
00–14 years	137	12.8 (9.1,15.0)	7.2 (6.0,8.4)
15 years and greater	524	8.3 (6.6,12.1)	8.1 (6.2,10.4)
**Contact with Female Index**	279	11.5 (7.7, 15.2)	7.7 (6.6,9.7)
00–14 years	108	14.0 (9.9,16.3)	7.3 (6.6,8.0)
15 years and greater	171	10.8 (7.0,13.9)	8.2 (6.6,10.9)
**Stratified by Age Index**			
**Contact with index 15–24 years**	296	11.3 (7.4,14.8)	7.9 (6.5,10.5)
00–14 years	95	14.2 (9.1,16.0)	7.4 (6.5,8.0)
15 years and greater	201	10.7 (6.9,13.6)	9.0 (6.5,11.2)
**Contact with index 25–44 years**	521	9.3 (6.7,13.1)	7.7 (6.3,10.0)
00–14 years	119	13.5 (9.4,15.6)	7.3 (6.0,8.6)
15 years and greater	402	8.3 (6.6,12.0)	7.9 (6.4,10.4)
**Contact with index 45 years and greater**	123	10.2 (7.0,12.8)	7.6 (6.1,9.6)
00–14 years	31	12.2 (6.9,14.0)	7.2 (6.1,7.8)
15 years and greater	92	10.0 (7.0,12.0)	7.7 (6.1,10.3)
**Stratified by Sex assortment**			
Female index with female contact	155	11.4 (7.2,15.1)	7.8 (6.8,10.3)
Female index with male contact	124	11.8 (8.2,15.4)	7.4 (6.4,9.1)
Male index with female contact	323	9.9 (6.9,13.3)	7.8 (6.0,10.7)
Male index with male contact	338	9.0 (6.7,12.5)	7.8 (6.5,9.7)

## Discussion

We identified two underlying constructs related to the social contact pattern between an infectious tuberculosis case and a contact from their social network. With these constructs, we developed a working definition of adequate contact between an infectious tuberculosis case and his or her contacts. The first factor characterized the setting and environment of interaction with the index cases, and the second factor described the social relationship between the index case and contact. These two factors explained 82% of the variance in the data, with the setting factor explaining the majority of it.

The setting score captured information about ventilation in the meeting place and about the nature of interaction in the venue. As for ventilation, inclusion of this variable provides strong content validity to the score because tuberculosis is transmitted through the airborne route in settings of shared airspace. Transmission is directly related to the concentration of bacteria in the inspired air and to the duration of exposure to contaminated air [38]. Ventilation reduces the concentration of organisms and would therefore be directly related to the likelihood of transmission. This score also captured the complex socio-spatial information about the physical proximity of cases and contacts in their patterns of sleeping and eating together.

The relationship score captured information about the intimacy, social and emotional closeness, of the relationship, especially as it relates to the health of the index case. This intimacy is shown by the trust an index case had for a contact. The score also reveals the reciprocity in the relationship, such that the contact provides care for the index case. This level of trust is not trivial given the stigma that is attached to tuberculosis in Africa because of its association with HIV [39]. The variable about means of transportation does not seem to fit the underlying construct, and indeed it had the lowest loading in the analysis. There is, however, a cultural interpretation that makes sense. Since transportation in Kampala is crowded and expensive, many residents walk to complete daily chores. When two individuals know one another, they are more likely to walk together. Extra-household contacts have low relationship scores whereas household contacts have higher relationship scores. These scores seem to vary from moderate to high, suggesting different levels of intimacy within the household.

Once the factor scores were estimated, we assessed construct validity by comparing scores according to relationship, age and sex, and household exposure. As for relationship, we postulated that family and relatives would have higher setting scores, and indeed they did. We observed that family members, especially spouses had the higher values when compared to community members, as expected. This factor may measure the nature of adequate contact among spouses that put them at higher risk for tuberculosis than other groups [30, 40]. However, it seems that even among these categories of contacts, there are degrees of closeness that should be considered. For instance, some siblings, relatives and children had setting scores as low as six units, but other family members had setting scores as high as 18 units. Thus, these scores could refine and further characterize the level of contact among contacts of an infectious case. We further postulated that household contacts would have higher setting scores, and as expected, they did. Finally, we evaluated age and sex interactions and found that younger contacts between birth and 14 years had higher setting scores with index cases between 15 and 45 years. This finding suggests age assortment of these child contacts with their parents or older siblings and partially explains the high risk for tuberculosis transmission to children in homes of infectious cases.

Regarding sex of contacts, women had higher setting scores than men. This finding was unexpected but seems to indicate the importance of the household in the transmission of *M. tuberculosis*. In a low- and middle-income settings, women tend to stay at home to fulfill their role as caregiver or because of limited opportunities for them in the formal job market [41]. Moreover, women in Uganda are reported to work 18% more than men in activities at home [42]. The relationship score was less variable across the different types of age and sex categories. There were no differences between female and male index cases. When assessing age assortment, we found that the relationship score was lower for contacts 0–14 years of age for all age groups of the index cases. Considering the nature of some of the questions that comprise this score the findings were as expected. Social intimacy and discussion of health issues seem to be topics that are more relevant to be shared within tuberculosis cases and adult contacts. The relationship score might be helpful to assess the level of social support that an index case receives from their social network. A systematic review of social network analyses in low- and middle- income settings has shown that behavior and health outcomes are associated with the structure and composition of these networks [43].

EFA has been criticized for identifying artificial factors that are not informative [44] to the underlying constructs being evaluated. One of the major strengths of our study is that we minimized this risk by conducting additional analyses that corroborate the robustness of our factors, as it has been recommended [45]. Our scores were consistent with other variables used to describe social network structure, e.g. nature of the relationship among contacts and case, age, and sex assortment.

There are several limitations of the study. First, the listed contacts in an index’s network may be incomplete. However, the median household size per index case was four, which is similar to the 3.7 reported among Kampala residents in the Uganda National Household Survey 2016/2017 report [46]. Second, there is a risk for recall and response bias because we collected self-reported data. Nevertheless, the nature of the questions and the high dispersion and variability of the factor scores suggest participants did not constrain their answers for social desirability [47]. Finally, there were some nominal variables that we had to exclude or recode as binary variables, thereby limiting the information in the analysis.

## Conclusion

In conclusion, our study identified two factors that can be used to assess adequate contact between tuberculosis cases and their contacts, explaining 82% of the variance in the observed variables. As a whole, these findings also confirm the complex and heterogeneous social mixing between cases and contacts [48]. In future studies, we will evaluate the criterion validity of these factors by relating them to the presence, or absence, of tuberculous infection among social networks of tuberculosis cases. Moreover, since social mixing differs across cultures [49], these factors could be evaluated in other contexts and other populations, including from middle- and high-burden countries.

## Supplementary information

**Additional file 1: Table S1.** Social network questionnaire conducted among 120 tuberculosis cases. **Table S2.** List of variables considered for the Exploratory Factor Analysis. **Table S3.** List of variables not considered for the Exploratory Factor Analysis. **Table S4.** Item analysis questionnaire social network form for the social contacts with complete social network data (n = 1154) and the contacts traced in the study that provided demographic data (*n* = 940). **Table S5.** Multivariate linear regression models for the association of setting score with characteristics of tuberculosis cases and social contacts. Overall and stratified analysis by household status and sex of contact. **Table S6.** Multivariate linear regression models for the association of relationship score with characteristics of tuberculosis cases and social contacts. Overall and stratified analysis by household status and sex of contact. **Figure S1.** Flow diagram of inclusion criteria for variables to be considered for the exploratory factor analysis. **Figure S2.** Eigenvalues of 13 components extracted during factor analysis. Factors with an eigenvalue ≥1 were retained in the model.

## Data Availability

The data that support the findings of this study are available on request from the senior author, CCW. The data are not publicly available due to containing information that could compromise the privacy of research participants.

## Abbreviations

EFA: Exploratory factor analysis
KMO: Kaiser-Meyer-Olkin
Bca: Adjusted bootstrap percentile
IQR: Interquartile range
