# Supplementary material for: Defining adequate contact for transmission of Mycobacterium tuberculosis in an African urban environment
Source: BMC Public Health. 2020 Jun 9;20:892. doi: 10.1186/s12889-020-08998-7 (PMC7285782; doi:10.1186/s12889-020-08998-7)
Supplement: Supplementary file 1 — Additional file 1: Table S1. Social network questionnaire conducted among 120 tuberculosis cases. Table S2. List of variables considered for the Exploratory Factor Analysis. Table S3. List of variables not considered for the Exploratory Factor Analysis. Table S4. Item analysis questionnaire social network form for the social contacts with complete social network data (n = 1154) and the contacts traced in the study that provided demographic data (n = 940). Table S5. Multivariate linear regression models for the association of setting score with characteristics of tuberculosis cases and social contacts. Overall and stratified analysis by household status and sex of contact. Table S6. Multivariate linear regression models for the association of relationship score with characteristics of tuberculosis cases and social contacts. Overall and stratified analysis by household status and sex of contact. Figure S1. Flow diagram of inclusion criteria for variables to be considered for the exploratory factor analysis. Figure S2. Eigenvalues of 13 components extracted during factor analysis. Factors with an eigenvalue ≥1 were retained in the model. [file 12889_2020_8998_MOESM1_ESM.docx]

SUPPLEMENTARY MATERIAL.

Table Legends

Table S1. Social network questionnaire conducted among 120 tuberculosis cases.

Table S2. List of variables considered for the Exploratory Factor Analysis.

Table S3. List of variables not considered for the Exploratory Factor Analysis.

Table S4. Item analysis questionnaire social network form for the social contacts with complete social network data (n=1,154) and the contacts traced in the study that provided demographic data (n=940).

Table S5. Multivariate linear regression models for the association of setting score with characteristics of tuberculosis cases and social contacts. Overall and stratified analysis by household status and sex of contact.

Table S6. Multivariate linear regression models for the association of relationship score with characteristics of tuberculosis cases and social contacts. Overall and stratified analysis by household status and sex of contact.

Table S1. Social network questionnaire conducted among 120 tuberculosis cases.

| **Question Number** | **Field Name** | **Brief Description** | **Response Option** |
| --- | --- | --- | --- |
| 1 | Nature | Nature of relationship with contact now | 01=Spouse  02=Child  03=Sibling  04=Friend  05=Stranger  06=Co-worker  07=Student colleague  08=Relative  09=Acquaintance  88=Other(specify) |
| 1  Others  (specify) | Rlnoth | Other specified relationship | Fill in |
| 2 | Newcont | Is contact new contact? | 01=Yes  02=No  77=Don’t Remember |
| 3 | Lgthknow | How long have you known contact | Fill in |
| 3 | Lgthtme | Unit of duration of knowing contact | 01=Days  02=Weeks  03=Months  04=Years |
| 4 | Lgcont | Duration of having name as contact | Fill in |
| 4 | Ltrel | Units of duration of having name as contact | 01=Days  02=Weeks  03=Months  04=Years |
| 5 | Bcough | Did you know name before you started to cough | 01=Yes  02=No  77=Don’t remember |
| 6 | Rlnchge | Has nature of relationship with contact change since the onset of cough | 01=Yes  02=No |
| 7 | Rln | Nature of relationship before the change | 01=Spouse  02=Co-worker  03=Student colleague  04=Relative  05=Friend  06=Acquaintance  88=Other(specify) |
| 7  Others  (Specify) | Pstrln | Other specified past relationship | Fill in |
| 8 | Meetfrq | Frequency of contact since the onset of cough? | 01=Increased  02=Decreased  03=Remained the same |
| 9 | well | How well does the informant know his contact? | 01=Very Well  02=Moderately well  03=Somewhat well  04=Not well  05=Almost do not know him/her |
| 10 | discuss | Does case discuss important life issues with contact? | 01=Yes  02=No  66=No response |
| 11 | confide | Does case confide with control? | 01=Yes  02=No  66=No Response |
| 12 | sharedtb | Shared TB diagnosis with contact? | 01=Yes  02=No |
| 13 | frqcont | Frequency of contact over the past 1 month | 01=None  02=Less than a day/week  03=1-3 days/week  04=4-6 days/week  05=Daily  77=Don’t recall |
| 14 | meettime | Duration of usual contact over the last one month | 01=Just a short time (Less than or equal to 1hr/day)  02=Part of the day (2-6 hrs./day)  03=Part of the day (7-12 hrs./day)  04=Most of the day(13-18hrs/day)  05=Over 18 hrs/day  77=I don’t recall  99=Not Applicable |
| 15a | meals | Shared meals with contact since the onset of cough? | 01=Yes  02=No  77=Don’t Remember |
| 15b | mealfrq | Frequency of sharing meals with contact | 01=< 1 day/week  02=1-3 days/week  03=4-6 days/week  04=Daily  77=Don’t recall  99=Not Applicable |
| 16a | sleep | Slept in same room with contact since the onset of cough? | 01=Yes  02=No |
| 16b | sleepfrq | Frequency of sleeping in the same room? | 01=< 1 day/week  02=1-3 days/week  03=4-6 days/week  04=Daily  77=Don’t recall  99=Not Applicable |
| 17a | bed | Slept on the same bed with contact since the onset of cough? | 01=Yes  02=No |
| 17b | bedfrq | Frequency of sleeping on same bed | 01=< 1 day/week  02=1-3 days/week  03=4-6 days/week  04=Daily  77=Don’t recall  99=Not Applicable |
| 18a | care | Provided care by the contact in the past 3 months | 01=Yes  02=No |
| 18b | carefrq | Frequency of care provided by contact in the past three months | 01=Less than a day/week  02=1-3 days/week  03=4-6 days/week  04=Daily  77=Don’t recall  99=Not Applicable |
| 19 | meetplc | Do you have a usual meeting place with contact? | 01=Yes  02=No |
| 20  Others  (specify) | meetusual | Place of usual meeting | 01=Your home  02=Friend’s home  03=Relative’s home  04=Work place  05=School  06=Worship center  07=Club/Association  08=Bar  09=Saloon  10=Gym  11=Trading center/Shop/Kiosk  12=In transit(specify)  88=Elsewhere(specify) |
| 20  Others  (specify) | intraspec | Specified usual transit meeting location | Fill in |
| 20 | meetoth | Specified other Meeting place with contact | Fill in |
| 21 | locdays | Frequency of meeting per week since the onset of work | 01=None  02=Less than a day/week  03=1-3 days/week  04=4-6 days/week  05=Daily  77=Don’t recall |
| 22 | loctime | Usual duration of meeting since the onset of cough | 01=Just a short time (Less than or equal to 1hr/day)  02=Part of the day (2-6 hrs./day)  03=Part of the day (7-12 hrs./day)  04=Most of the day(13-18hrs/day)  05=Over 18 hrs/day  77=I don’t recall |
| 23  Others  (specify) | mostmeet | Most recent meeting location with contact | 1=Your home  2=Friend’s home  3=Relative’s home  4=Work place  5=School  6=Worship center  7=Club/Association  8=Bar  9=Saloon  10=Gym  11=Trading center/Shop/Kiosk  12=In transit (specify)  88=Elsewhere (specify) |
| 23  Others  (specify) | transpec | Other specified usual transit of meeting |  |
| 23 | mostoth | Other location of most recent meeting | Fill in |
| 24 | timespent | Time spent in the most recent meeting with contact | 01=Just a short time (Less than or equal to 1hr/day)  02=Part of the day (2-6 hrs./day)  03=Part of the day (7-12 hrs./day)  04=Most of the day(13-18hrs/day)  05=Over 18 hrs/day  77=I don’t recall |
| 25 | meet | Meet contact indoors or outdoors? | 01=Mostly Indoors  02=Mostly Outdoors  03=Equally inside and outside |
| 26 | vent | Nature of ventilation at usual meeting place | 01=Poor: Completely enclosed place (All windows and doors are closed)  02=Minimal: Partially enclosed (Some windows and/or doors closed)  03=Fair: Structure has a roof, enclosed in four walls with large opening typical of a retail shop  04=Full: Completely outdoor, under a tree, under a roof supported by poles  77=Don’t know |
| 27 | pple | Meet other people in addition to contact at usual location? | 01=Yes  02=No |
| 28 | pplenum | Number of other people met in addition to contact | Fill in |
| 29 | locoth | Other location of meeting | 01=Yes  02=No |
| 30 | othloc | Frequency of meeting at the other location | 01=None  02=< 1 day/week  03=1-3 days/week  04=4-6 days/week  05=Daily  77=Don’t recall |
| 31 | trans | Means of transport used together with contact since the onset of cough | 01=Motor bike  02=Bodaboda  03=Private vehicle  04=Taxi  05=Lorry  06=Bus  07=Train  08=Plane  09=Boat  10=None  11=Others(specify) |
| 31  Others  (specify) | transoth | Other specified means of transportation | Fill in |
| 32 | means | Means of transportation used most often with contact | 01=Motor bike  02=Bodaboda  03=Private vehicle  04=Taxi  05=Lorry  06=Bus  07=Train  08=Plane  09=Boat  10=None  88=Others(specify) |
| 32  Others  (specify) | meansoth | Other specified means of transportation used most often with contact | Fill in |
| 33 | cough | Does contact have a cough? | 01=Yes  02=No  77=Don’t know |
| 34 | tb | Does contact have a TB? | 01=Yes  02=No  77=Don’t know |

Table S2. List of variables considered for the Exploratory Factor Analysis. Description, variable name and final recoding.

| **Number** | **Brief Description** | **Variable name** | **Final recoding** |
| --- | --- | --- | --- |
| 1 | How long have you known contact? (years) | known | 1= Up to 2 years  2= 2-4 years  3= 4-10 years  4= More than 10 years |
| 2 | Frequency of contact since the onset of cough? | meetingfrequency | **1**='Decreased'  **2**='Same frequency'  **3**='Increased'; |
| 3 | How well does the informant know his contact? | Well | **1**='Not well/almost do not know'  **2**='Somewhat well'  **3**='Moderately well'  **4**='Very well'; |
| 4 | Does case discuss and confide important life issues with contact? | Trust | **1**='No discuss nor confide'  **2**='Discuss but not confide'  **3**='Discuss and confide'; |
| 5 | Shared TB diagnosis with contact? | sharedtb | 1=No  2=Yes |
| 6 | Frequency and duration of contact over the past 1 month | Hourscat | **1**=' hours <= 3.5/week'  **2**='hours 3.5-28 hrs/week'  **3**='hours >28-66.5/week'  **4**='>66.5 hrs/week/week'; |
| 7 | Frequency of sharing meals with contact | Meals | **1**='Not shared meals'  **2**='Shared meals, less than a day per week'  **3**='Shared meals 1-3 days/week'  **4**='Shared meals 4-6/week'  **5**='Shared meals daily'; |
|  |  |  |  |
| 8 | Frequency of sleeping in the same room and bed? | Bed | **1**='No slept same room nor bed'  **2**='Slept same room, but not same bed'  **3**='Slept same room and same bed, not daily'  **4**='Slept same room and same bed, daily'; |
|  |  |  |  |
| 9 | Frequency of care provided by contact in the past three months | care | **1**='No care by contact'  **2**='Provided care, less than a day per week'  **3**='Provided care 1-3 days/week'  **4**='Provided care 4-6/week'  **5**='Provided care daily'; |
|  |  |  |  |
| 10 | Place of usual meeting with contact | Place | 1=Not the house of TB case  2=House TB case  . =Missing |
| 11 | Meet contact indoors or outdoors? | Meet | **1**='Mostly meeting outdoors'  **2**='Equally indoors/outdoors'  **3**='Mostly meeting indoors'; |
| 12 | Nature of ventilation at usual meeting place | vent | **1**='Full ventilation'  **2**='Fair ventilation'  **3**='Minimal ventilation'  **4**='Poor ventilation'; |
| 13 | Number of other people met in addition to contact at usual location. | Peoplecat | **1**='< 2 persons/meeting'  **2**='2-4 persons/meeting'  **3**='5-6 persons/meeting'  **4**='>6 persons/meeting'; |
|  |  |  |  |
| 14 | Means of transportation used most often with contact | transportation | **1**='None/walking'  **2**='Another type of transportation'; |
| 15 | Does contact have a cough? | cough | 1=No/Don’t know  2=Yes |

Table S3. List of variables not considered for the Exploratory Factor Analysis. Description, variable name, final recoding and Reason to be not considered.

| **Number** | **Brief Description** | **Recoded name** | **Final recoding** | **Reason not considered** |
| --- | --- | --- | --- | --- |
| 1 | Does contact have a TB? | tb | 1=No/Don’t know  2=Yes | Low variation (98% no/don’t know, 2% yes) |
| 2 | Did you know name before you started to cough | bcough | 1=No/Don’t remember  2=Yes | Low variation (97% yes) |
| 3 | Is this contact a new contact? | TBN_newcont |  | Low variation (99% no) |
| 4 | Has nature of relationship with contact change | rlnchge | 01=Yes  02=No | Low variation (99% no) |
| 5 | Nature of relationship before the change | rln | 01=Spouse  02=Co-worker  03=Student colleague  04=Relative  05=Friend  06=Acquaintance  88=Other(specify) | Low variation (99.8% missing, because answer to previous question). Also, categorical variable. |
| 6 | How long have you have this person as contact (years) | contacttime | Continuous variables (in years) | Response equal to answer for ‘How long have you known contact (years)?’-in its continuous variable form |
| 7 | Nature of relationship with contact now | TBN_nature | 01=Spouse  02=Child  03=Sibling  04=Friend  05=Stranger  06=Co-worker  07=Student colleague  08=Relative  09=Acquaintance  10=Neighbor  88=Other(specify) | Categorical variable |
| 8 | Most recent meeting location with contact | TBN_mostmeet | **1**="Your home"  **2**="Friend’s home"  **3**="Relative’s home"  **4**="Work place"  **5**="School"  **6**="Worship center"  **7**="Club/Association/Bar/Saloon/Gym"  **8**="Bar"  **9**="Saloon"  **10**="Gym"  **11**="Trading center/Shop/Kiosk"  **12**="In transit"  **13**="Neigbourhood"  **88**="Elsewhere(specify)"  **.** ="Missing" | Correlation: 0.92 with TBN_most usual variable, which was the original question for the recoded “Place” variable. |
| 9 | Time spent in the most recent meeting with contact | TBN_timespent | 01=Just a short time (Less than or equal to 1hr/day)  02=Part of the day (2-6 hrs./day)  03=Part of the day (7-12 hrs./day)  04=Most of the day(13-18hrs/day)  05=Over 18 hrs/day  . =Missing | Correlation: 0.93 with Hourscat |
| 10 | Other location of meeting | TBN_locoth | 01=Yes  02=No | Not informative enough. |
| 11 | Frequency of meeting at the other location | TBN_othloc | 01=None  02=< 1 day/week  03=1-3 days/week  04=4-6 days/week  05=Daily  77=Don’t recall |  |
| 12 | Means of transport used together with contact since the onset of cough | TBN_Trans | 01=Motor bike  02=Bodaboda  03=Private vehicle  04=Taxi  05=Lorry  06=Bus  07=Train  08=Plane  09=Boat  10=None  11=Others(specify) | Not informative enough. |
| 13 and 14* | Frequency and duration of meeting per week since the onset of work | Hoursloccat    *This variable represented two questions in the original questionnaire.  1) Frequency and 2) Duration. | **1**=' hours <= 3.5/week'  **2**='hours 3.5-28 hrs/week'  **3**='hours >28-66.5/week'  **4**='>66.5 hrs/week/week'; | Correlation: 0.97 with Hourscat |

Table S4. Item analysis questionnaire social network form for the social contacts with complete social network data (n=1,154) and the contacts traced in the study that provided demographic data (n=940). Included categorical variables and recoded variables considered for the factor analysis.

| **Variable** | **1154 contacts with complete social network data** | | **940 traced contacts with other variables collected** | |
| --- | --- | --- | --- | --- |
|  | **N** | **Percent** | **n** | **Percent** |
| **Nature of relationship with tuberculosis case^1^** |  |  |  |  |
| Spouse | 34 | 2.9 | 25 | 2.7 |
| Child | 140 | 12.1 | 123 | 13.1 |
| Sibling | 151 | 13.1 | 117 | 12.4 |
| Friend | 342 | 29.6 | 276 | 29.4 |
| Co-workers | 86 | 7.5 | 67 | 7.1 |
| Oher relative | 215 | 18.6 | 175 | 18.6 |
| Neighbor | 100 | 8.7 | 82 | 8.7 |
| Other | 86 | 7.5 | 75 | 8.0 |
| **Length knowing the contact** |  |  |  |  |
| Less than 2 years | 428 | 37.1 | 360 | 38.3 |
| 2-4 years | 206 | 17.9 | 167 | 17.8 |
| 5-6 years | 236 | 20.5 | 200 | 21.3 |
| More than 6 years | 284 | 24.6 | 213 | 22.7 |
| **Frequency of meeting since onset cough** |  |  |  |  |
| Decreased | 63 | 5.5 | 45 | 4.8 |
| Same frequency | 1029 | 89.2 | 852 | 90.6 |
| Increased | 62 | 5.4 | 43 | 4.6 |
| **Hours spent per week with contact** |  |  |  |  |
| Less than 3.5 hours/week | 333 | 28.9 | 272 | 28.9 |
| Between 3.5-28 hours/week | 461 | 39.9 | 374 | 39.8 |
| Between 28-66.5 hours/week | 272 | 23.6 | 212 | 22.6 |
| Greater 66.5 hours/week | 88 | 7.6 | 82 | 8.7 |
| **Location of usual meeting^2^ (detailed responses)** |  |  |  |  |
| Home of tuberculosis case | 645 | 55.9 | 525 | 55.9 |
| Friend’s home | 65 | 5.6 | 56 | 6.0 |
| Relative’s home | 72 | 6.2 | 55 | 5.9 |
| Work place | 202 | 17.5 | 164 | 17.4 |
| Bar | 40 | 3.5 | 35 | 3.7 |
| Trading center/Shop/Kiosk | 54 | 4.7 | 45 | 4.8 |
| Elsewhere | 76 | 6.6 | 60 | 6.4 |
| **Location of usual meeting^2^ (binary for EFA)** |  |  |  |  |
| Outside home of tuberculosis case | 509 | 44.1 | 415 | 44.1 |
| Home of tuberculosis case | 645 | 55.7 | 525 | 55.9 |
| **Ventilation place of meeting** |  |  |  |  |
| Full ventilation | 555 | 48.1 | 462 | 49.1 |
| Fair ventilation | 218 | 18.9 | 172 | 18.3 |
| Minimal ventilation | 183 | 15.9 | 145 | 15.4 |
| Poor ventilation | 198 | 17.2 | 161 | 17.1 |
| **Indoor or outdoor meeting** |  |  |  |  |
| Mostly meeting outdoors | 550 | 47.7 | 445 | 48.4 |
| Equally indoors/outdoors | 289 | 25.0 | 241 | 25.6 |
| Mostly meeting indoors | 315 | 27.3 | 244 | 26.0 |
| **Number of other people met in addition to contact†** |  |  |  |  |
| < 2 persons/meeting | 345 | 29.9 | 274 | 29.1 |
| 2-4 persons/meeting | 435 | 37.7 | 353 | 37.6 |
| 5-6 persons/meeting | 255 | 22.1 | 216 | 23.0 |
| >6 persons/meeting | 119 | 10.3 | 97 | 10.3 |
| **Sleeping conditions** |  |  |  |  |
| No slept in same room, nor bed | 903 | 78.3 | 738 | 78.5 |
| Slept same room, but not same bed | 169 | 14.6 | 137 | 14.6 |
| Slept same room and same bed, not daily | 21 | 1.8 | 16 | 1.7 |
| Slept same room and same bed, daily | 61 | 5.3 | 49 | 5.2 |
| **Meals** |  |  |  |  |
| Not shared meals | 447 | 38.7 | 371 | 39.5 |
| Shared meals, less than a day per week | 106 | 9.2 | 79 | 8.4 |
| Shared meals 1-3 days/week | 174 | 15.1 | 134 | 14.3 |
| Shared meals 4-6 days/week | 66 | 5.7 | 58 | 6.2 |
| Shared meals daily | 361 | 31.3 | 298 | 31.7 |
| **Case trusts contact** |  |  |  |  |
| No discuss nor confide | 480 | 41.6 | 404 | 43.0 |
| Discuss but not confide | 364 | 31.5 | 292 | 31.1 |
| Discuss and confide | 310 | 26.9 | 244 | 26.0 |
| **Shared TB diagnosis** |  |  |  |  |
| No | 643 | 55.7 | 532 | 56.6 |
| Yes | 511 | 44.3 | 408 | 43.4 |
| **Care by contact** |  |  |  |  |
| No care by contact | 971 | 84.1 | 792 | 84.3 |
| Care provided, less than a day per week | 42 | 3.6 | 33 | 3.5 |
| Provided care 1-3 days/week | 55 | 4.8 | 39 | 4.1 |
| Provided care 4-6 days/week | 16 | 1.4 | 14 | 1.5 |
| Provided care daily | 70 | 6.1 | 62 | 6.6 |
| **How well does the case knows contact** |  |  |  |  |
| Not well/almost do not know | 18 | 1.6 | 16 | 1.7 |
| Somewhat well | 159 | 13.8 | 132 | 14.0 |
| Moderately well | 269 | 23.2 | 220 | 23.4 |
| Very well | 709 | 61.4 | 572 | 60.9 |
| **Means of transportation used most often with contact. None (walking) versus a type of transportation.** |  |  |  |  |
| None/walking | 925 | 80.2 | 763 | 81.2 |
| Another type of transportation | 229 | 19.8 | 177 | 18.8 |
| **Known if contact has cough** |  |  |  |  |
| No | 1082 | 93.8 | 875 | 93.1 |
| Yes | 72 | 6.2 | 65 | 6.9 |

^1^The variable ‘‘Nature of the relationship between case and contact’ (spouse, child, sibling, friend, co-worker, relative, neighbor, other) was excluded for EFA but the descriptive analysis is reported.

^2^The categorical variable ‘Location of usual place of meeting’ (Home case, friend’s home, relative’s home, work place, bar, trading center/shop/kiosk, elsewhere) was recoded as a binary variable (Home case, outside home of tuberculosis), and included in the EFA as a dichotomous variable but the descriptive analysis of the original variables is reported here.

Table S5. Multivariate linear regression models for the association of setting score with characteristics of tuberculosis cases and social contacts. Overall and stratified analysis by household status and sex of contact.

|  | Dependent variable: Setting score | | | | |
| --- | --- | --- | --- | --- | --- |
|  | Results from five regression models (M1-M5) β (SE) | | | | |
|  | Overall | Subsets of the overall population | | | |
| Model and population | M1. All | M2. Only HH women | M3. Only EHH women | M4. Only HH men | M5. Only EHH men |
| Observations | 940 | 194 | 284 | 156 | 306 |
| Type of contact (household vs extra household) | 6.206^***^ |  |  |  |  |
|  | (0.158) |  |  |  |  |
| Age of contact (years) | 0.003 | -0.004 | 0.025^**^ | -0.025^**^ | 0.005 |
|  | (0.005) | (0.011) | (0.011) | (0.011) | (0.011) |
| Age of index (years) | 0.016^**^ | 0.018 | 0.010 | -0.007 | 0.046^***^ |
|  | (0.008) | (0.018) | (0.013) | (0.018) | (0.015) |
| Se of contact (men vs women) | 0.115 |  |  |  |  |
|  | (0.147) |  |  |  |  |
| Sex of index (men vs women) | -0.773^***^ |  |  |  |  |
|  | (0.174) |  |  |  |  |
| Sex assortment (yes vs no) |  | 1.160^***^ | 0.904^***^ | -0.690^*^ | -0.398 |
|  |  | (0.373) | (0.316) | (0.403) | (0.339) |
| Constant | 8.092^***^ | 13.660^***^ | 6.780^***^ | 15.317^***^ | 7.032^***^ |
|  | (0.294) | (0.683) | (0.561) | (0.565) | (0.530) |
| Adjusted R^2^ | 0.647 | 0.037 | 0.033 | 0.040 | 0.025 |

M#=Number of models. HH=Only household contacts. EHH=Only extra household contacts.

^*^p^**^p^***^p<0.01

Table S6. Multivariate linear regression models for the association of relationship score with characteristics of tuberculosis cases and social contacts. Overall and stratified analysis by household status and sex of contact.

|  | Dependent variable: Relationship score | | | | |
| --- | --- | --- | --- | --- | --- |
|  | Results from five regression models (M1-M5) β (SE) | | | | |
|  | Overall | Subsets of the overall population | | | |
| Model and population | M1. All | M2. Only HH women | M3. Only EHH women | M4. Only HH men | M5. Only EHH men |
| Observations | 940 | 194 | 284 | 156 | 306 |
| Type of contact (household vs extra household) | 3.057^***^ |  |  |  |  |
|  | (0.139) |  |  |  |  |
| Age of contact (years) | 0.077^***^ | 0.115^***^ | 0.055^***^ | 0.099^***^ | 0.038^***^ |
|  | (0.005) | (0.010) | (0.009) | (0.010) | (0.009) |
| Age of index (years) | -0.010 | -0.017 | 0.008 | -0.016 | -0.004 |
|  | (0.007) | (0.015) | (0.011) | (0.016) | (0.013) |
| Se of contact (men vs women) | -0.110 |  |  |  |  |
|  | (0.129) |  |  |  |  |
| Sex of index (men vs women) | 0.150 |  |  |  |  |
|  | (0.153) |  |  |  |  |
| Sex assortment (yes vs no) |  | -0.108 | 0.427 | 0.792^**^ | 0.391 |
|  |  | (0.326) | (0.265) | (0.344) | (0.295) |
| Constant | 5.584^***^ | 8.427^***^ | 5.413^***^ | 7.738^***^ | 6.301^***^ |
|  | (0.258) | (0.596) | (0.472) | (0.483) | (0.462) |
| Adjusted R^2^ | 0.394 | 0.426 | 0.104 | 0.407 | 0.062 |

M#=Number of models. HH=Only household contacts. EHH=Only extra household contacts.

^*^p^**^p^***^p<0.01

SUPPLEMENTARY MATERIAL.

Figure Legends

Figure S1. Flow diagram of inclusion criteria for variables to be considered for the exploratory factor analysis.

Figure S2. Eigenvalues of thirteen components extracted during factor analysis.

Factors with an eigenvalue ≥ 1 were retained in the model.

Exploratory Analysis of each question

Questions Excluded (n=14)

Not enough variation: 05

Correlation: >0.90 with another variable: 04

Response equal to answer of other question: 01

Categorical variable: 01

Not informative enough: 03

Questions Included (n=20)

Used as single variable (n=10)

Used in combination with another variable (From n=10 to n=5)

15 recoded variables considered for exploratory factor analysis

34 questions in questionnaire from 1,179 contacts

Figure S1. Flow diagram of inclusion criteria for variables to be considered for the exploratory factor analysis.


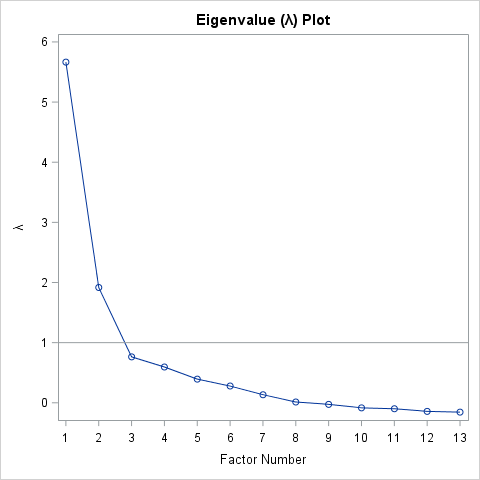


Figure S2. Eigenvalues of thirteen components extracted during factor analysis.

Factors with an eigenvalue ≥ 1 were retained in the model.
